# Lumbar Radicular Pain Response to First Injection with Non-particulate Steroid

**DOI:** 10.7759/cureus.7104

**Published:** 2020-02-26

**Authors:** Jason Lipetz, Perry Zelinger, Myriam Kline, Nadeen Chahine, Ona Bloom

**Affiliations:** 1 Physical Medicine and Rehabilitation, Hofstra-Northwell School of Medicine/ Long Island Spine Rehabilitation Medicine, Great Neck, USA; 2 Physical Medicine and Rehabilitation, Rusk Rehabilitation, NYU Langone Health, New York, USA; 3 Biostatistics Unit, Feinstein Institute for Medical Research, Northwell Health, Manhasset, USA; 4 Orthopedic Surgery, Columbia University, New York, USA; 5 Molecular Medicine, Physical Medicine and Rehabilitation, The Feinstein Institute for Medical Research/Hofstra Northwell School of Medicine, Manhasset, USA

**Keywords:** pain, lumbar radiculopathy, epidural injections, disc herniation, stenosis

## Abstract

Introduction

Recent studies on the use of transforaminal epidural steroid injection (TFESI) to treat lumbar radicular pain have highlighted controversies pertaining to the choice of corticosteroid agent utilized in lumbosacral TFESI, in terms of both safety and efficacy. The primary objective was to characterize the radicular pain response after a first transforaminal injection with dexamethasone. The secondary objective was to document the response of those who failed to respond to a dexamethasone injection when particulate steroid was utilized for a second injection.

Methods

It was a retrospective study of 94 consecutive patients undergoing transforaminal injection for lumbosacral radicular pain. At two-week follow-up, patients rated their pain response on a clinically oriented five-point survey. First injection non-responders were given a second injection with particulate steroid and again completed the survey.

Results

Approximately one-third (N = 31/94) of patients received no meaningful relief from a single injection with dexamethasone. No patients achieved lasting and complete pain relief after a single injection. Of initial non-particulate steroid non-responders, approximately two-thirds (N = 19/28) demonstrated a notable or complete response to a second injection with particulate steroid.

Conclusions

We are now able to better inform patients with regard to their anticipated pain response to an initial dexamethasone injection. Only one-third of patients realized more significant and lasting relief after a single injection. Of those who did not demonstrate a more meaningful response, a second injection with particulate steroid resulted in more pronounced pain relief in two-thirds of patients.

## Introduction

Lumbosacral radicular pain is a commonly encountered clinical presentation that can be associated with extreme discomfort, disability, and fear amongst patients. Most commonly, such radicular pain complaints arise from either an acute disc herniation or more chronic degenerative stenosis. Fortunately, the natural history of these cases is often benign and without resultant long-term disability [1]. When more conservative treatment measures fail to provide adequate relief, transforaminal epidural steroid injection (TFESI) may be an effective means of providing both pain relief and avoiding surgical intervention [2,3]. Most of the prior literature examining the use of TFESI has typically employed the use of particulate corticosteroids [4,5].

Recent studies on the use of TFESI to treat lumbar radicular pain have highlighted controversies pertaining to the choice of corticosteroid agent utilized in lumbosacral TFESI, in terms of both safety and efficacy. While the overall complication rate is extremely low, case reports of permanent and devastating neurologic injury following lumbosacral TFESI have been reported [6-10]. One mechanism of proposed injury is arterial occlusion resulting from crystalloid steroid particles during inadvertent intravascular injection. While particulate steroid particles can be larger than red blood cells and have the propensity to form even larger aggregates, dexamethasone, the only commercially available non-particulate steroid, is completely soluble [11,12]. An animal study demonstrating ischemic neural injury in pigs following vertebral artery injection of particulate steroid, but no such response following injection with dexamethasone, appears to support this concept [13]. Interestingly, a recent case report of a conus medullaris infarct following a lumbar TFESI with dexamethasone suggests alternative and non-occlusive means of vascular and neurological injury may exist [14].

Several well-designed studies have now compared the efficacy of TFESI when performed with particulate and non-particulate corticosteroids. While several studies have shown non-inferiority of dexamethasone, one study revealed superior pain relief with the injection of particulate triamcinolone, and another demonstrated a trend toward fewer injections required to achieve similar relief when particulate steroids were injected [15-19]. In light of safety concerns, published interdisciplinary guidelines, and FDA recommendations and labeling, many more TFESI procedures are now being performed with dexamethasone [20-21].

The purpose of this study was to characterize radicular pain responses to an initial injection with dexamethasone. This information might then better inform clinical expectations for both the interventionalist and the patient, following what can be a long awaited and initial injection procedure. The secondary aim of this study was to characterize the responses of a subset of patients who failed to achieve meaningful pain relief with an initial injection of dexamethasone and then went on to receive a subsequent injection of triamcinolone.

## Materials and methods

Methods

This retrospective chart review was performed with the approval of the local Institutional Review Board. All injections were performed by one interventional physiatrist in a private physiatric practice in a metropolitan suburb between June 2015 and June 2016. Data was collected retrospectively via electronic medical records. Patients were included in the study if they were over 18 years of age and had a clinical history, physical exam, and imaging studies consistent with prevailing lumbosacral radicular greater than axial pain, as diagnosed by a single fellowship-trained interventional physiatrist with 18 years of clinical experience. The exclusion criteria were: prior injection at the same practice, prior history of lumbar spinal surgery, workers compensation or no fault insurance, and back pain greater than leg pain. Consecutive patients (N = 94) meeting these criteria underwent lumbosacral transforaminal epidural steroid injection with dexamethasone for lumbosacral radicular pain.

At a two-week follow-up appointment, patients were asked to rate their pain response on a custom survey. The choices on the survey were: “No improvement at all”, “Improved for a day or two but now no improvement at all”, “There is some but not a marked improvement from the first injection”, “There is a definite and notable improvement after the first injection”, or “I am pain free after the first injection and do not feel that I need another.” To facilitate data analysis, we later assigned a numerical value of 0-4 according to the survey responses (Table 1). Patients who responded “0” or “1” to the survey were considered non-responders to the first injection and were given a second injection with triamcinolone. This subset of patients was asked to fill out the pain survey again at the next two-week follow-up. Those patients who reported a numerical response of “2” or “3” to the first injection with dexamethasone were no longer asked to complete response surveys and continued with dexamethasone containing injections. Clinical and demographic information was also obtained and analyzed: diagnosis (acute disc herniation vs. chronic spinal stenosis), age, gender, BMI, current smoker (Y/N), diabetes (Y/N), and pain greater or less than one year’s duration.-

**Table 1:** Pain response survey completed by patients at two-week follow-up appointment and corresponding numerical assignment

Self-reported pain survey response	Assigned numerical value (0-4)
No improvement at all	0
Improved for a day or two but now no improvement at all	1
There is some but not a marked improvement from the first injection	2
There is a definite and notable improvement after the first injection	3
I am pain free after the first injection and do not feel that I need another	4

All injections were performed by a single interventional physiatrist according to standard International Spine Intervention Society guidelines [22]. The patient was positioned prone on the fluoroscopy table and prepared in a sterile manner. A skin wheal was raised with 1% lidocaine and added bicarbonate in the paraspinal region overlying the targeted pedicle. Utilizing a single needle technique and an oblique visualization, a 22G spinal needle was advanced to the “6 o’clock” position of the targeted pedicle. Proper needle positioning was then confirmed under anteroposterior (AP) visualization. Contrast medium (Omnipaque; GE Healthcare, Princeton, NJ) was injected (~5.0 mL) through microbore tubing to achieve a 0.5 mL spread of contrast under live fluoroscopic visualization. The exiting nerve root was clearly outlined with both cephalad spread beneath the pedicle and flow along the exiting spinal nerve. A test dose of 1% lidocaine was then injected with patient monitoring and questioning for any adverse effect for 120 seconds. Dexamethasone (10 mg/mL, Decadron; Merck, Whitehouse Station, NJ) was then injected (~1 mL for single-level or 1.5 mL for two-level injections), followed by 1% lidocaine (0.5 mL). For second injections with triamcinolone acetonide (40 mL/mg, Kenalog; Bristol-Myers Squibb, Princeton, NJ), 1 mL was used for single-level and 1.5 mL was used for two-level injections.

Data analysis

The primary objective was to determine the percentage of consecutive patients who failed to realize meaningful pain relief after a single injection with the soluble steroid dexamethasone. We analyzed potential differences in therapeutic responses between patients presenting with radicular pain of stenotic vs. discogenic origin. We also examined the effects of multiple demographic factors on potential differences in pain responses after treatment. The secondary objective was to determine the likelihood of a more clinically meaningful response in patients who were treated with a second injection of a particulate steroid after a first injection with dexamethasone failed to provide symptom relief.

The frequencies and percentages for each of our clinical and demographic variables were determined. Chi-square and Fisher’s exact testing were utilized in the assessment of categorical variables. The Kruskal-Wallis test was used in the assessment of continuous data. Spearman correlation coefficient was employed to assess the relationship between continuous variables and first injection response.

## Results

Consecutive patients (N = 94) who had an initial lumbosacral transforaminal epidural steroid injection with dexamethasone for radicular pain were included in this study. Demographic and clinical information for the patients is shown below (Table 2). There was no association between baseline diagnosis, i.e. discogenic (herniated disc) vs. stenotic radicular pain and any included demographic and clinical variables. The mean age of herniated disc patients was 56, while that of stenosis patients was 72 years.

**Table 2:** Baseline demographic and clinical data for 94 consecutive patients with lumbosacral radicular pain

Variable	Total patients = 94 N (%) or Mean (±SD)
Male	49 (52.13%)
Female	45 (47.87%)
Age	66 (±14) Range 20-89
Discogenic	34 (36.17%)
Spinal Stenosis	60 (63.83%)
Duration of symptoms <1 year	60 (63.83%)
Duration of symptoms >1 year	34 (36.17%)
Diabetes	7 (7.53%)
Current Smoker	6 (6.52%)
BMI	27.99 (±5.9) Range 19.2-51

Patients were asked to rate their pain after treatment using a custom survey (Table 1). Of the patients who received a 1st injection with dexamethasone, nine (9.6%) reported a response score of “0”, 22 (23.4%) reported a response graded as “1”, 31 (33%) were rated a “2”, and 32 (34.0%) were rated a “3”. There were no patients who rated themselves a “4”, or “pain free” after one injection with dexamethasone (Table 3).

**Table 3:** First and second injection scores The combined 31 patients who scored a "0" or "1" on first injection were selected to receive a 2nd injection with triamcinolone. Of the 31, two were lost to follow up and one had a prior adverse reaction to triamcinolone resulting in a total second injection (n = 28).

Injection Score	First injection (n = 94) with dexamethasone n (%)	Second injection (n = 28) with triamcinolone n (%)
0	9 (9.57%)	2 (7.1%)
1	22 (23.40%)	3 (10.7%)
2	31 (32.98%)	4 (14.3%)
3	32 (34.04%)	17 (60.7%)
4	0 (0%)	2 (7.1%)

Among all baseline clinical and demographic data, there was only a significant association between the symptom relief score after the first injection and gender (p = 0.04). More females than expected scored “0” on first injection (eight females vs. one male). There was no statistically significant association between symptom relief scores after first injection and age, BMI, diagnosis, smoking status, symptom duration, or diabetes (P > 0.05 for all variables) (Table 4). These analyses were performed both for individual numerical variables, as well as collapsed categories of relative injection failures (“0” and “1” combined) and relative injection successes (“2” and “3” combined), with no difference in statistical significance observed.

**Table 4:** First injection scores by variable

	0	1	2	3	4	P value
n (%) or mean (SD)	n = 9	n = 22	n = 31	n = 32	n = 0	
Discogenic	4 (11.76%)	7 (20.59%)	9 (26.47%)	4 (41.18%)	0	0.59
Spinal Stenosis	5 (8.33%)	15 (25%)	22 (36.67%)	18 (30%)	0	
Male	1 (2.04%)	10 (20.41%)	19 (38.78%)	19 (38.78%)	0	0.040
Female	8 (17.78%)	12 (26.67%)	12 (26.67%)	13 (28.89%)	0	
Mean Age (SD)	59.56 (17.73)	69 (14.28)	67.97 (12.66)	64.53 (14.97)	N/A	0.34
BMI (SD)	25.66 (4.63)	28.8 (5.21)	28.66 (7.09)	27.39 (5.44)	N/A	0.52
Smoker	0 (0%)	2 (33.33%)	1 (16.67%)	3 (50%)	0	0.76
Duration of Symptoms <1 yr	3 (5%)	17 (28.33%)	19 (31.67%)	21 (35%)	0	0.15
Duration of Symptoms >1 yr	6 (17.65%)	5 (14.71%)	12 (35.29%)	11 (32.35%)	0	
Diabetes	0 (0%)	1 (14.29%)	2 (28.57%)	4 (57.14%)	0	0.68

Of the 94 patients who received a first injection, 31 scored a “0” or “1,” signifying a group that was clinically non-responsive in achieving meaningful pain relief and thus were selected to receive a 2nd injection using particulate steroid. Within this group of non-responders to the first injection, two patients were lost to follow up and one patient refused a 2nd injection with triamcinolone due to a poor tolerance to this corticosteroid in the past, resulting in 28 patients who received a second injection with triamcinolone. Of these 28 patients, two (7.1%) rated themselves a “0”, three (10.7%) were rated “1”, four (14.3%) were rated “2”, 17 (60.7%) were rated “3”, and two (7.1%) rated themselves a “4” (Table 3).

## Discussion

The goal of this study was to characterize radicular pain responses to a first injection with the non-particulate steroid dexamethasone. Our study found that one-third of patients did not realize any meaningful pain relief by two weeks after a single injection. Another two-thirds of patients realized ‘some’ or ‘notable’ relief two weeks after the first injection: with 33% exhibiting “some but not a marked” level of relief and another 34% exhibiting “definite and notable” relief. The likelihood of treatment failure in 33% of patients did not seem to be affected by age, smoking or diabetic status, chronicity of symptoms, or stenotic vs. discogenic origin of root compression. However, female patients were more likely to have no meaningful pain relief compared to male patients.

Studies of gender differences in experimental pain indicate greater pain sensitivity among females than males [23]. This is consistent with studies in mice that have identified a biological basis for gender dependent pain, namely that different immune cells and inflammatory pathways are active during pain hypersensitivity in male vs. female mice [24]. Moreover, sex-specific response to pain treatments that occur may be related to immune cell contributions. This sex difference will be important to consider when developing treatments for pain and other neurological disorders involving immune system pathways.

Adopting change in one’s treatment paradigm can be difficult. This is particularly true when a practice has realized two decades of reasonable success in treating radicular pain with the use of injected particulate steroids and without a single major adverse event. Over the course of that time and over thirty thousand unique patient encounters, we have trialed different particulate and non-particulate steroids during transforaminal injection therapy for cervical and lumbar radicular pain. Our experience has been that more particulate steroids tend to be more effective, with fewer overall non-surgical treatment failures and with fewer injections required to realize non-surgical success. This study was conducted on nearly 100 consecutive patients at a time in our practice evolution where we, in accordance with our interpretation of the then current literature, guidelines, and medical legal environment, were employing dexamethasone as a first line injection for lumbar radicular pain. Particulate corticosteroid injection was to be reserved only for those who were labeled a non-particulate injection non-responder. It is the response to a first injection with dexamethasone for lumbosacral radicular pain which is the focus of this report.

For the patient presenting with pronounced radicular pain, the first injection in a potential series is arguably the most critical for several reasons. These patients have often failed a combination of analgesics, NSAIDS, oral steroids, therapy, chiropractic care, and/or acupuncture and are attempting to avoid surgery. The first injection offers hope that pain relief is near. At the same time, anxiety levels of a patient are usually highest preceding the first injection, a unique experience filled with the “unknown” for the novice patient. If the first injection offers no symptom relief at all, or minimal measurable benefit, the likelihood may be reduced for that patient to continue a required series of three or even four injections. In addition, insurance companies may ask for accurate documentation of relief if additional injections in a series are to be approved.

We chose a treatment response scale for this study which best reflected how we communicate with our patients on a daily basis and in determining if further injections are indicated. This is the scale used within the study site for clinical purposes. We have found this pain survey to be easily interpretable by both clinicians and patients. It was for this reason that we did not employ more continuous or numeric variables as outcome measures for this report. We find that numeric score reports can vary from visit to visit and over time, without a corroborative change in overall daily pain status. Numeric pain scores are therefore not utilized in our practice as the primary determinant driving the potential for a continued series of spinal interventions. For this reason, initial pain scores prior to the first injection were also not documented in this series. What each patient had in common was debilitating radicular pain arising from a stenotic or more focal disc compressive correlate. Each patient had failed a reasonable trial of less interventional treatment measures.

Historically, in our treatment of these patients with radicular pain, a small subset would realize complete relief with a single injection utilizing either particulate betamethasone or, perhaps more commonly, triamcinolone. A more pronounced pain response to particulate steroid might be theorized to arise from a less soluble medication having a more sustained local therapeutic effect. It is our observation that transforaminal epidural injection with particulate triamcinolone, particularly at higher dosages (i.e., greater than 40 mg), is also more likely to result in both lasting systemic therapeutic and less desirable adverse responses. We question if this systemic effect of triamcinolone does in fact contribute to the pain response realized by those treated for radicular pain through epidural injections. Following dexamethasone injection, we have also more commonly observed a described level of initial and dramatic relief followed by a return to baseline symptoms two or three days later. A similar dexamethasone injection response was described by 23.4% (n = 22) of patients in the current study. In this study, not a single patient experienced complete pain relief two weeks after their first injection with dexamethasone. Therefore, all patients proceeded to a second steroid injection.

Our study found that one-third (33%) of patients did not realize any meaningful pain relief two weeks after a single injection with dexamethasone. This initial lack of a treatment response may be consistent with several well designed studies that revealed a greater number of injections with non-particulates were required to achieve similar degrees of lasting symptom relief than in those treated with particulate steroids [16]. In addition to documenting an absence of “one and done” scenarios, this study provides us with additional data to substantiate our discussions with patients in terms of their first injection response expectations.

It is important that this potential lack of a lasting first injection response be faithfully communicated to patients, so that hope is not lost for ultimate therapeutic success following a more complete series of injections. This point, combined with the now available and more robust and well-designed multicenter trials examining the efficacy of dexamethasone injections, might also be highlighted in communications with insurance carriers who might otherwise not allow for second injections.

In this study, another third of patients realized “some but not a marked” level of relief two weeks after the first injection. The remaining third of patients described “definite and notable” relief. As a result of these data, at the study site this characterization of initial responses is now used to guide treatment expectations for patients. Patients are advised that they are very unlikely to realize the complete pain relief they desire with a single injection alone. In this study, patients who failed to realize any relief after a first injection of dexamethasone had the opportunity to have a second injection, using a particulate or non-particulate steroid. Within the process of obtaining informed treatment consent, the practice experience, the state of the literature, evolving treatment guidelines, and the incidence and severity of potential complications were described to each patient.

Of those patients who chose to receive a second injection of a particulate steroid (triamcinolone), 18% still did not achieve lasting symptom relief at a subsequent two-week follow-up evaluation. The other 82% of patients did achieve lasting pain relief at the two-week visit following a single injection with triamcinolone. Of particular note, 68% realized at least “definite and notable” relief. Seven percent were “pain free” and did not feel additional injection therapy was necessary. No patients who failed to realize lasting benefit from the first injection with dexamethasone chose to proceed with a second injection with the same steroid, so we are unaware of what their response might have been. A choice was given. Patients remained in a good deal of pain, and when offered the educated opportunity, chose to switch steroid. This lack of an ability to compare second injection responses between particulate and non-particulate steroids is a weakness of our study. This second injection response data may be subsequently extracted from previous well-designed studies which completed injection series utilizing dexamethasone alone [15-19].

Multiple prospective outcome studies support the use of dexamethasone for all transforaminal injections [15-19]. The medical legal environment and described potentially devastating complications which can follow particulate corticosteroid use also provide data supporting non-particulate steroids for all transforaminal injections [7-10, 20-21]. The previous studies of dexamethasone also reveal that a greater number of injections may be required to achieve the same therapeutic benefit as when a particulate steroid is injected [16]. With each injection, there is a statistical increase in the likelihood of a procedural complication or adverse response. The incidence of any, let alone more devastating, complications following transforaminal injection therapy performed by experienced interventionalists utilizing accepted procedural technique is exceedingly low [6]. A recent study also highlights that the use of dexamethasone alone does not completely remove the possibility of a more tragic vascular type complication during a lumbar transforaminal injection approach [14]. This study suggests that a third of patients in pain are not likely to realize more lasting relief after a single injection with dexamethasone. This, combined with a greater number of injections required to realize a desired outcome, may result in a prolonged period of suffering, disability, lost income, and more patients proceeding to surgery. The surgical failure and complication rate must also be recognized along with the percentage of patients requiring repeat surgical procedures [25,26]. Regardless of steroid injected, we must all advise our patients that a percentage with ongoing radicular pain will ultimately require surgical decompression to realize lasting relief [27].

Considering the observations of this paper, clinical experience, the medical literature, and medical legal environment, injection protocols for lumbar radicular pain at the study site practice have evolved. We now use dexamethasone as a first line transforaminal injection approach for all patients with lumbosacral radicular pain. Treatment with dexamethasone without pain relief is not considered a treatment failure until no meaningful response has been realized after two injections with dexamethasone. This assumes the insurance carrier will allow us to proceed without more supportive documentation after an initial injection. Those failed patients after two injections, who wish to continue injection therapy, are offered an interlaminar injection approach with a particulate steroid. Following this paradigm, we have observed several patients who realize a “notable” or “pain free” response after one interlaminar injection. Only in those patients who refuse surgery and whose anatomy or pathology does not allow for an interlaminar injection, a particulate transforaminal injection is offered with informed consent.

The science of pain medicine is inexact. We are only just beginning to understand the multifaceted origins of pain and the infinitely varied human responses. The treatment of radicular pain and the spectrum of therapeutic response observed are no exception. Our goal must always remain “to do good, and not to do harm” with the admittedly limited knowledge base we currently possess. The findings of this study will enable us to better educate our patients in terms of their likely response to the first (and often most anticipated) steroid injection to treat radicular pain. Many questions are not addressed by this study. This includes the persistent question of what is the most appropriate and evidence-based next step for those patients with radicular pain who fail to realize any lasting relief after a single injection with dexamethasone.

## Conclusions

Approximately one-third of patients with lumbosacral radicular pain received no meaningful relief from a first injection of the soluble steroid dexamethasone. Another third of patients realized “some but not a marked” level of relief two weeks after the first injection, while the final third described “definite and notable” relief. No patients in this study were able to achieve complete and lasting pain relief without undergoing a second injection procedure. The likelihood of treatment failure was significantly affected by patient gender, with female patients more likely to report no meaningful pain relief compared to male patients.
